# Association of Vitamin D Receptor Gene Polymorphisms with Colorectal Cancer in a Saudi Arabian Population

**DOI:** 10.1371/journal.pone.0155236

**Published:** 2016-06-16

**Authors:** Khayal A. Alkhayal, Zainab H. Awadalia, Mansoor-Ali Vaali-Mohammed, Omar A. Al Obeed, Alanoud Al Wesaimer, Rabih Halwani, Ahmed M. Zubaidi, Zahid Khan, Maha-Hamadien Abdulla

**Affiliations:** 1 Colorectal Research Chair, Department of Surgery, King Khalid University hospital, College of medicine, King Saud University, Riyadh, Saudi Arabia; 2 Prince Sultan Military Medical City, Department of internal medicine, Riyadh, Saudi Arabia; 3 Prince Naif Center for Immunology Research and Asthma Research Chair, Department of Pediatrics, College of Medicine, King Saud University, Riyadh, Saudi Arabia; 4 Genome Research Chair, Department of Biochemistry, College of Science, King Saud University, Riyadh, Saudi Arabia; University of Birmingham, UNITED KINGDOM

## Abstract

**Background:**

Vitamin D, causally implicated in bone diseases and human malignancies, exerts its effects through binding to the vitamin D receptor (VDR). VDR is a transcription factor modulating the expression of several genes in different pathways. Genetic variants in the VDR gene have been associated with several cancers in different population including colorectal cancer.

**Objective:**

To assess the association of VDR gene polymorphisms in relation with colorectal cancer (CRC) in a Saudi population.

**Methods:**

The polymorphisms of VDR gene (BsmI, FokI, ApaI and TaqI) were analyzed by the polymerase chain reaction amplification of segments of interest followed by Sanger sequencing. One hundred diagnosed CRC patients and 100 healthy control subjects that were age and gender matched were recruited.

**Results:**

We did not observe significant association of any of the four VDR polymorphisms with colorectal cancer risk in the overall analysis. Although not statistically significant, the AA genotype of BsmI conferred about two-fold protection against CRCs compared to the GG genotype. Stratification of the study subjects based on age and gender suggests statistically significant association of CRC with the ‘C’ allele of ApaI in patients **>**57 years of age at disease diagnosis and BsmI polymorphism in females. In addition, statistically significant differences were observed for the genotypic distributions of VDR-BsmI, ApaI and TaqI SNPs between Saudi Arabian population and several of the International HapMap project populations.

**Conclusion:**

Despite the absence of correlation of the examined VDR polymorphisms with CRCs in the combined analysis, ApaI and BsmI loci are statistically significantly associated with CRC in elderly and female patients, respectively. These findings need further validation in larger cohorts prior to utilizing these SNPs as potential screening markers for colorectal cancers in Saudi population.

## Introduction

Colorectal cancer (CRC) is the fourth leading cause of cancer-related mortality worldwide with its incidence increasing rapidly in recent years [1]. The incidence of CRC is on the rise in the Kingdom of Saudi Arabia. CRC presentation is at a younger age in Saudis, especially in women [2]. Besides genetics, environmental factors may contribute to the development of the disease. In 1980 Garland and Garland hypothesized that higher incidence rates for CRC in areas with low sunlight exposure might be attributable to lower levels of vitamin D [3]. Deficiency of vitamin D is a common occurrence in Saudi Arabia, especially among young women and is becoming a major public health concern. Several factors that contribute to the high prevalence of vitamin D deficiency have been proposed which include insufficient nutritional intake along with inadequate exposure to sunlight, abnormality in vitamin D absorption and malnutrition [4].

Vitamin D is involved in biological processes such as bone metabolism, cell proliferation and differentiation. An inverse relationship between serum vitamin D and the incidence of cancer has been suggested [5]. It has been reported that Vitamin D reduces epithelial cell proliferation and promote differentiation in human colon derived cells as well as in experimental animal studies [6–8]. A higher serum level of 25-OH vitamin D has been associated with decreased colorectal adenoma risk [9, 10]. Vitamin D deficiency has been associated with breast cancer [11], ovarian cancer [12] and prostate cancer [13–14].

Vitamin D (1, 25(OH)_2_D_3,_ calcitriol) acts via binding to an intracellular vitamin D receptor (VDR) in target tissues [15]. VDR is located on chromosome 12q12-14 and consists of two promotor regions, eight protein coding exons (2–9) and six untranslated exons (1a-1f) [16]. More than sixty SNPs of the VDR gene located in the promoter region have been discovered in relation to cancer occurrence and prognosis [15, 17]. ApaI (rs7975232), TaqI (rs731236), BsmI (rs1544410) and FokI (rs10735810) are functional VDR SNPs that affect VDR gene expression and mRNA stability [9].

Recently, several epidemiologic studies reported the association between various VDR genetic variants and CRC with contradictory results [18–24] and significant associations between VDR gene SNPs with the risk of CRC has been presented [18, 20, 21, 22, and 24]. In contrast, other studies reported no association [19, 22]. Furthermore, the association between VDR gene polymorphisms and the risk of CRC may be associated with adiposity [23].

The aim of this study was to investigate four SNPs (FokI, BsmI, TaqI and ApaI) within the VDR gene in Saudi patients with CRC versus healthy controls. The selection of these SNPs were based on the common VDR SNPs sites that were examined in other populations in previous genetic epidemiological studies.

## Materials and Methods

### Study population

One hundred sporadic CRC patients (age from 20–80 years old) who underwent operations for CRC at King Khalid University Hospital (KKUH) in the period between 2013 and 2014, and 100 healthy subjects of equivalent age range were recruited for blood donation at the KKUH, Riyadh, Saudi Arabia. All adult donors were requested to sign a fully informed and written consent, approved by the Ethics Review Committee of the KKUH. We received 95% response rate from patients and controls that were requested to participate in the study. This study was approved by the Ethical Committee of the king Saud University.

### DNA extraction

Peripheral blood was extracted and DNA was purified from 3 mL of whole blood, using a QIAamp DNA Blood Mini Kit (QIAGEN Sciences, Valencia, CA, USA), following the manufacturer's instructions. DNA concentrations were determined spectrophotometrically on NanoDrop 8000 (Thermo Scientific, USA), and the purity determined by using standard A260/A280 and A260/A230 ratios. Samples were stored in -80°C until used.

### Genotyping

Genotyping for FokI (rs2228570), BsmI (rs1544410), TaqI (rs731236) and ApaI (rs7975232) polymorphisms in the VDR gene was performed by PCR amplification, followed by sequencing. PCR amplification of the region of the gene encompassing the variants was done using DNA polymerase, 5XFIREPol Master Mix (Solis BioDyne, Estonia, Europe) and specific primers. The following primers were used **FokI**: 5’-CATGTATGAGGGCTCCGAAG (right) and 5’- ACCTTGCTTCTTCTCCCTCC- (left); **BsmI**: 5’- AGGACAAAGACCTGCTGAGG (right) and 5’- TCACTGCACATTGCCTCCAA (left); **TaqI**: 5’- CTGAGAGCTCCTGTGCCTTC (right) and 5’-AAGGGGCGTTAGCTTCATGC (left); and **ApaI**: 5’- GGTATCACCGGTCAGCAGTC (right) and 5’- CGTTAGCTTCATGCTGCACT (left). Cycle sequencing of the purified PCR fragments was performed using the Big Dye Terminator kit by capillary electrophoresis (Applied Biosystems, Sequencer model 3730xl). Genotyping analysis was performed with the Sequencing Analysis software version 5.2 (Applied Biosystems, CA, USA).

### Statistical analysis

In this case-control study, tests for association and deviation from Hardy-Weinberg equilibrium were performed using the web based program http://ihg.gsf.de/cgi-bin/hw/hwa1.pl as done previously [25]. Chi-square values, odds ratios (OR), 95% confidence intervals (CI) and p-values were calculated to compare the distribution of genotype and allele frequencies between the CRC patients and healthy controls. Pair-wise chi-square analysis was performed in a 3 x 2 contingency table to assess the difference in the distribution of genotypes between Saudi Arabians and the International HapMap project populations. The genotype counts for the International HapMap populations were obtained from the HapMap project database release #28 (http://hapmap.ncbi.nlm.nih.gov). A p-value of <0.05 was considered as significant.

## Results

### Association between VDR SNPs and CRC risk

In this case-control study, a total of 200 subjects were examined for four SNPs in the VDR gene to determine the risk of predisposition to colorectal cancer. The study group comprised 100 histopathologically confirmed CRCs and 100 age and gender matched normal healthy controls from Saudi Arabian ethnicity. The age of the CRC patients were between 20 and 80 with the median age of 57.5 years while the controls aged between 21 and 81 with the median age being 57.5 years. The clinicopathological characteristics of CRC patients has been reported earlier [25]. Briefly, 64% of the cases were males and 36% were females. Majority of the primary tumors were located in the colon (85%) while 15% of the patients had tumor in the rectum. According to the Union internationale contre le cancer (UICC) clinical staging, 11%, 58% and 17% patients were categorized as stage I, stage II and stage III, respectively, while 14% patients had metastatic disease. Histological grading as per UICC classified 97% of the cases as G2.

The distribution of genotypes at SNPs loci rs7975232 and rs731236 in the CRC group and that of SNPs rs1544410 and rs7975232 in the control group deviated from the Hardy-Weinberg equilibrium (Table 1). Genotypes of rs1544410 and rs2228570 in the CRC patients and that of rs2228570 and rs731236 in the controls were in accordance with the Hardy-Weinberg equilibrium (Table 1). The allelic and genotype frequencies of the analyzed SNPs in the VDR gene in CRCs and normal population are shown in Table 2. In the overall analysis, none of the four genetic variants in the VDR gene were found to be significantly associated with risk of developing colorectal cancer. Although the AA genotype of rs1544410 conferred about two-fold decreased risk of developing CRCs compared to the GG genotype, this association was not statistically significant (OR = 0.492, χ^2^ = 3.14, p = 0.07629) (Table 2).

**Table 1:** Test for deviation from Hardy-Weinberg equilibrium.

SNP ID	Genotype	CRCn (frequency)	HWE P-value	Controls n (frequency)	HWE P-value
rs1544410	GG	47 (0.47)	0.212481	38 (0.38)	0.043336
	GA	39 (0.39)		39 (0.39)	
	AA	14 (0.14)		23 (0.23)	
rs2228570	CC	59 (0.59)	0.496616	58 (0.58)	0.771326
	CT	34 (0.34)		37 (0.37)	
	TT	07 (0.07)		05 (0.05)	
rs7975232	AA	40 (0.40)	0.000033	45 (0.45)	1.032e-07
	AC	29 (0.29)		23 (0.23)	
	CC	31 (0.31)		32 (0.32)	
rs731236	CC	16 (0.16)	0.047860	16 (0.16)	0.248002
	CT	36 (0.36)		41 (0.41)	
	TT	48 (0.48)		43 (0.43)	

CRC. Colorectal Cancer; HWE. Hardy-Weinberg equilibrium

**Table 2:** Distribution of VDR SNPs genotype and allele frequencies in colorectal cancer cases and control population.

SNP ID	Genotype	CRC n (frequency)	Controls n (frequency)	OR (95% CI)	χ^2^ -Value	P*- Value	P_Corr_- Value
rs1544410	GG	47 (0.47)	38 (0.38)	Ref			
	GA	39 (0.39)	39 (0.39)	0.809 (0.437–1.497)	0.46	0.49884	1
	AA	14 (0.14)	23 (0.23)	0.492 (0.223–1.084)	3.14	0.07629	0.30516
	**Allele**						
	G	133 (0.665)	115 (0.575)	Ref			
	A	67 (0.335)	85 (0.425)	0.682 (0.454–1.023)	3.44	0.06371	0.25484
rs2228570	CC	59 (0.59)	58 (0.58)	Ref			
	CT	34 (0.34)	37 (0.37)	0.903 (0.501–1.630)	0.11	0.73559	1
	TT	07 (0.07)	05 (0.05)	1.376 (0.413–4.585)	0.27	0.60182	1
	**Allele**						
	C	152 (0.76)	153 (0.765)	Ref			
	T	48 (0.24)	47 (0.235)	1.028 (0.649–1.629)	0.01	0.90647	1
rs7975232	AA	40 (0.40)	45 (0.45)	Ref			
	AC	29 (0.29)	23 (0.23)	1.418 (0.709–2.838)	0.98	0.32240	1
	CC	31 (0.31)	32 (0.32)	1.090 (0.568–2.092)	0.07	0.79597	1
	**Allele**						
	A	109 (0.545)	113 (0.565)	Ref			
	C	91 (0.455)	87 (0.435)	1.084 (0.731–1.609)	0.16	0.68736	1
rs731236	CC	16 (0.16)	16 (0.16)	Ref			
	CT	36 (0.36)	41 (0.41)	0.878 (0.385–2.004)	0.10	0.75728	1
	TT	48 (0.48)	43 (0.43)	1.116 (0.499–2.499)	0.07	0.78903	1
	**Allele**						
	C	68 (0.34)	73 (0.365)	Ref			
	T	132 (0.66)	127 (0.635)	1.116 (0.740–1.682)	0.27	0.60077	1

CRC. Colorectal Cancer; OR 95% CI. Odds Ratio and 95% Confidence Interval; P_Corr_. Bonferroni corrected p-value.

* P < 0.05 was considered significant

### Age at disease diagnosis and gender based correlation of VDR SNPs and CRC

To determine whether genetic variants in the VDR gene have any influence on the age of onset of CRC, patients were grouped according to the median age at disease diagnosis as ≤57 (n = 50) and >57 (n = 50) years. The distribution of genotypes and allelic frequencies in the CRC patient group were compared to the age matched controls and are depicted in Table 3. Only the rs7975232 exhibited statistically significant association with CRCs based on the allelic model in patients of age group >57 years. Individuals with the C allele at rs7975232 have about two-fold higher risk of developing CRC compared to those having the A allele (OR = 1.778, χ^2^ = 4.00, p = 0.04554). Additionally, it was observed that the CC homozygosity compared to the AA homozygosity of rs7975232 impart about two-fold greater risk of acquiring CRCs, however, this association did not reach statistical significance (OR = 2.273, χ^2^ = 2.90, p = 0.08860) (Table 3). The other SNPs in the VDR gene that were examined were not associated significantly with colorectal cancer cases in either age group.

**Table 3:** Distribution of VDR SNPs genotype and allele frequencies in colorectal cancer cases and control population based on age.

SNP ID	Genotype	CRCn (frequency)	Controls n (frequency)	OR (95% CI)	χ^2^ -Value	P*- Value	P_Corr_- Value
≤57							
rs1544410	GG	21 (0.42)	18 (0.36)	Ref			
	GA	24 (0.48)	22 (0.44)	0.935 (0.398–2.199)	0.02	0.87768	1
	AA	05 (0.10)	10 (0.20)	0.429 (0.123–1.488)	1.83	0.17662	0.70648
	**Allele**						
	G	66 (0.66)	58 (0.58)	Ref			
	A	34 (0.34)	42 (0.42)	0.711 (0.401–1.262)	1.36	0.24384	0.97536
rs2228570	CC	33 (0.66)	34 (0.68)	Ref			
	CT	14 (0.28)	13 (0.26)	1.110 (0.454–2.713)	0.05	0.81968	1
	TT	03 (0.06)	03 (0.06)	1.030 (0.194–5.476)	0.00	0.97206	1
	**Allele**						
	C	80 (0.80)	81 (0.81)	Ref			
	T	20 (0.20)	19 (0.19)	1.066 (0.529–2.146)	0.03	0.85835	1
rs7975232	AA	21 (0.42)	20 (0.40)	Ref			
	AC	17 (0.34)	09 (0.18)	1.799 (0.653–4.958)	1.30	0.25413	1
	CC	12 (0.24)	21 (0.42)	0.544 (0.213–1.389)	1.63	0.20127	0.80508
	**Allele**						
	A	59 (0.59)	49 (0.49)	Ref			
	C	41 (0.41)	51 (0.51)	0.668 (0.382–1.168)	2.01	0.15597	0.62388
rs731236	CC	06 (0.12)	07 (0.14)	Ref			
	CT	24 (0.48)	23 (0.46)	1.217 (0.355–4.170)	0.10	0.75400	1
	TT	20 (0.40)	20 (0.40)	1.167 (0.333–4.089)	0.06	0.80956	1
	**Allele**						
	C	36 (0.36)	37 (0.37)	Ref			
	T	64 (0.64)	63 (0.63)	1.044 (0.587–1.857)	0.02	0.88323	1
>57							
rs1544410	GG	26 (0.52)	20 (0.40)	Ref			
	GA	15 (0.30)	17 (0.34)	0.679 (0.274–1.680)	0.70	0.40133	1
	AA	09 (0.18)	13 (0.26)	0.533 (0.190–1.493)	1.45	0.22816	0.91264
	**Allele**						
	G	67 (0.67)	57 (0.57)	Ref			
	A	33 (0.33)	43 (0.43)	0.653 (0.367–1.160)	2.12	0.14517	0.58068
rs2228570	CC	26 (0.52)	24 (0.48)	Ref			
	CT	20 (0.40)	24 (0.48)	0.769 (0.341–1.733)	0.40	0.52644	1
	TT	04 (0.08)	02 (0.04)	1.846 (0.310–11.011)	0.46	0.49608	1
	**Allele**						
	C	72 (0.72)	72 (0.72)	Ref			
	T	28 (0.28)	28 (0.28)	1.000 (0.539–1.854)	0.00	1.00000	1
rs7975232	AA	19 (0.38)	25 (0.50)	Ref			
	AC	12 (0.24)	14 (0.28)	1.128 (0.425–2.990)	0.06	0.80887	1
	CC	19 (0.38)	11 (0.22)	2.273 (0.877–5.891)	2.90	0.08860	0.3544
	**Allele**						
	A	50 (0.50)	64 (0.64)	Ref			
	C	50 (0.50)	36 (0.36)	1.778 (1.009–3.131)	4.00	**0.04554**	0.18216
rs731236	CC	10 (0.20)	09 (0.18)	Ref			
	CT	12 (0.24)	18 (0.36)	0.600 (0.188–1.913)	0.75	0.38640	1
	TT	28 (0.56)	23 (0.46)	1.096 (0.381–3.150)	0.03	0.86535	1
	**Allele**						
	C	32 (0.32)	36 (0.36)	Ref			
	T	68 (0.68)	64 (0.64)	1.195 (0.665–2.147)	0.36	0.55045	1

CRC. Colorectal Cancer; OR 95% CI. Odds Ratio and 95% Confidence Interval; P_Corr_. Bonferroni corrected p-value.

* P < 0.05 was considered significant and are depicted in bold

The distribution of genotype and allele frequencies of the four VDR gene polymorphisms segregated according to gender are presented in Table 4. In the male patients, none of the VDR gene polymorphisms were significantly associated with CRC. However, the rs1544410 was found to associated significantly with CRCs in females (OR = 0.217, χ^2^ = 4.21, p = 0.04021). The AA homozygous women are 4.6 fold less likely to get CRCs relative to those having GG homozygosity of rs1544410. In the allelic model as well similar association with CRCs was observed in female patients where the A allele imparted significant protective effect (Table 4). The other three SNPs, rs2228570, rs7975232 and rs731236 were not found to be associated with colorectal cancers in female patients.

**Table 4:** Distribution of VDR SNPs genotype and allele frequencies in colorectal cancer cases and control population based on gender.

SNP ID	Genotype	CRC n (frequency)	Controls n (frequency)	OR (95% CI)	χ^2^ -Value	P*- Value	P_Corr_- Value
**Male**							
rs1544410	GG	28 (0.43)	27 (0.42)	Ref			
	GA	26 (0.40)	23 (0.35)	1.090 (0.504–2.356)	0.05	0.82644	1
	AA	11 (0.17)	15 (0.23)	0.707 (0.276–1.811)	0.52	0.46949	1
	**Allele**						
	G	82 (0.631)	77 (0.592)	Ref			
	A	48 (0.369)	53 (0.408)	0.850 (0.516–1.401)	0.40	0.52464	1
rs2228570	CC	38 (0.585)	37 (0.57)	Ref			
	CT	23 (0.354)	24 (0.37)	0.933 (0.450–1.935)	0.03	0.85242	1
	TT	04 (0.061)	04 (0.06)	0.974 (0.227–4.184)	0.00	0.97140	1
	**Allele**						
	C	99 (0.76)	98 (0.754)	Ref			
	T	31 (0.24)	32 (0.246)	0.959 (0.544–1.691)	0.02	0.88492	1
rs7975232	AA	26 (0.40)	26 (0.40)	Ref			
	AC	20 (0.31)	17 (0.26)	1.176 (0.506–2.738)	0.14	0.70602	1
	CC	19 (0.29)	22 (0.34)	0.864 (0.380–1.961)	0.12	0.72595	1
	**Allele**						
	A	72 (0.554)	69 (0.531)	Ref			
	C	58 (0.446)	61 (0.469)	0.911 (0.559–1.485)	0.14	0.70882	1
rs731236	CC	12 (0.185)	09 (0.138)	Ref			
	CT	23 (0.354)	24 (0.369)	0.719 (0.255–2.026)	0.39	0.53158	1
	TT	30 (0.461)	32 (0.492)	0.703 (0.259–1.907)	0.48	0.48792	1
	**Allele**						
	C	47 (0.36)	42 (0.32)	Ref			
	T	83 (0.64)	88 (0.68)	0.843 (0.505–1.408)	0.43	0.51342	1
**Female**							
rs1544410	GG	19 (0.543)	11 (0.314)	Ref			
	GA	13 (0.371)	16 (0.457)	0.470 (0.166–1.334)	2.03	0.15376	0.61504
	AA	03 (0.086)	08 (0.228)	0.217 (0.047–0.993)	4.21	**0.04021**	0.16084
	**Allele**						
	G	51 (0.73)	38 (0.54)	Ref			
	A	19 (0.27)	32 (0.46)	0.442 (0.218–0.896)	5.21	**0.02242**	0.08968
rs2228570	CC	21 (0.60)	21 (0.60)	Ref			
	CT	11 (0.31)	13 (0.37)	0.846 (0.310–2.312)	0.11	0.74456	1
	TT	03 (0.09)	01 (0.03)	3.000 (0.288–31.225)	0.91	0.33885	1
	**Allele**						
	C	53 (0.76)	55 (0.786)	Ref			
	T	17 (0.24)	15 (0.214)	1.176 (0.534–2.592)	0.16	0.68729	1
rs7975232	AA	14 (0.40)	19 (0.54)	Ref			
	AC	09 (0.26)	06 (0.17)	2.036 (0.588–7.052)	1.28	0.25856	1
	CC	12 (0.34)	10 (0.29)	1.629 (0.549–4.828)	0.78	0.37773	1
	**Allele**						
	A	37 (0.53)	44 (0.63)	Ref			
	C	33 (0.47)	26 (0.37)	1.509 (0.769–2.964)	1.44	0.23088	0.92352
rs731236	CC	04 (0.114)	07 (0.20)	Ref			
	CT	13 (0.371)	17 (0.486)	1.338 (0.322–5.564)	0.16	0.68816	1
	TT	18 (0.514)	11 (0.314)	2.864 (0.679–12.079)	2.13	0.14452	0.57808
	**Allele**						
	C	21 (0.30)	31 (0.443)	Ref			
	T	49 (0.70)	39 (0.557)	1.855 (0.925–3.718)	3.06	0.08027	0.32108

CRC. Colorectal Cancer; OR 95% CI. Odds Ratio and 95% Confidence Interval; P_Corr_. Bonferroni corrected p-value.

* P < 0.05 was considered significant and are depicted in bold

### Comparison of VDR SNPs genotypes distribution between KSA and International HapMap project populations

The genotypic distribution of the three polymorphic loci, rs1544410, rs7975232 and rs731236 in the VDR gene for which the data were available from the International HapMap project populations (http://hapmap.ncbi.nlm.nih.gov/) were compared to Saudi Arabian control population. Pairwise chi-square test showed that the distribution of the three genotypes of rs1544410 was significantly different between the Saudi Arabian population (KSA) and those from the HapMap project populations except Utah residents with Northern and Western European ancestry (CEU); Maasai in Kinyawa, Kenya (MKK) and Tuscan in Italy (TSI) (Table 5). The genotype frequencies of rs7975232 for all the eleven populations included in the HapMap project were significantly different compared to our population. In the case of rs731236 SNP, the genotypes distribution in the KSA population used in this study were similar to that found in five International HapMap project populations namely, African ancestry in Southwest USA (ASW); CEU; Gujarati Indians in Houston, Texas (GIH); Luhya in Webuye, Kenya (LWK) and TSI. Statistically significant difference was observed for the rs731236 genotypes in KSA population compared to the Han Chinese in Beijing, China (CHB); Chinese in Metropolitan Denver, Colorado (CHD); Japanese in Tokyo, Japan (JPT); Mexican ancestry in Los Angeles, California (MEX); MKK and Yoruban in Ibadan, Nigeria (YRI) included in the HapMap project (Table 5).

**Table 5:** Comparison of VDR SNPs genotypes between KSA and International HapMap project populations.

SNP ID	Population (n)	Genotype count			χ^2^ -Value	P*- Value
rs1544410		GG	GA	AA		
	KSA (100)	38	39	23	-	-
	ASW (57)	30	27	0	15.5093	**0.000429**
	CEU (113)	39	49	25	0.4409	0.802158
	CHB (137)	127	10	0	84.4513	**< 0.00001**
	CHD (109)	100	9	0	69.3461	**< 0.00001**
	GIH (101)	30	57	14	6.5006	**0.038764**
	JPT (113)	89	24	0	46.4313	**< 0.00001**
	LWK (109)	63	41	5	17.4544	**0.000162**
	MEX (58)	34	22	2	12.3049	**0.002128**
	MKK (154)	61	72	21	3.9431	0.139244
	TSI (101)	34	49	18	1.9634	0.374671
	YRI (147)	80	54	13	11.6238	**0.002992**
rs7975232		AA	AC	CC		
	KSA (100)	45	23	32	-	-
	ASW (57)	20	31	6	18.1764	**0.000113**
	CEU (113)	42	45	26	7.0747	**0.02909**
	CHB (137)	11	52	74	43.7886	**< 0.00001**
	CHD (109)	12	46	51	30.7909	**< 0.00001**
	GIH (101)	21	61	19	29.2272	**< 0.00001**
	JPT (113)	12	51	50	32.9805	**< 0.00001**
	LWK (110)	59	42	9	19.9099	**0.000047**
	MEX (58)	9	33	16	21.4717	**0.000022**
	MKK (156)	70	71	15	25.0427	**< 0.00001**
	TSI (102)	37	46	19	11.7422	**0.00282**
	YRI (145)	52	70	23	18.075	**0.000119**
rs731236		CC	CT	TT		
	KSA (100)	16	41	43	-	-
	ASW (56)	3	22	31	4.5202	0.104341
	CEU (113)	25	49	39	2.0962	0.350599
	CHB (136)	0	8	128	76.7708	**< 0.00001**
	CHD (109)	0	10	99	56.6451	**< 0.00001**
	GIH (101)	7	47	47	4.1037	0.128495
	JPT (112)	0	23	89	36.5306	**< 0.00001**
	LWK (109)	9	41	59	4.0898	0.129391
	MEX (57)	2	22	33	6.6571	**0.035844**
	MKK (156)	22	91	43	8.0206	**0.018128**
	TSI (101)	18	49	34	1.8758	0.391453
	YRI (147)	8	67	72	7.5697	**0.022712**

**KSA:** Kingdom of Saudi Arabia; **ASW:** African ancestry in Southwest USA; **CEU:** Utah residents with Northern and Western European ancestry from the CEPH collection; **CHB:** Han Chinese in Beijing, China; **CHD:** Chinese in Metropolitan Denver, Colorado; **GIH:** Gujarati Indians in Houston, Texas; **JPT:** Japanese in Tokyo, Japan; **LWK:** Luhya in Webuye, Kenya; **MEX:** Mexican ancestry in Los Angeles, California; **MKK:** Maasai in Kinyawa, Kenya; **TSI:** Tuscan in Italy; **YRI:** Yoruban in Ibadan, Nigeria; * P < 0.05 was considered significant and are depicted in bold

## Discussion

Deficiency of vitamin D has been implicated in bone diseases, metabolic syndromes and human malignancies. 1, 25(OH)_2_D_3_ bring about its antitumorigenic effects by promoting cellular differentiation while decreasing cell growth, proliferation, invasion and metastasis [26]. Since vitamin D acts through its binding to VDR, suboptimal responsiveness of the VDR can be manifested as vitamin D deficiency. Polymorphisms in VDR gene have been significantly associated with various cancers including prostate, breast, skin and colorectal [27]. Several investigations have focused on the association of genetic variants in the VDR gene and predisposition of colorectal cancers in different populations [28–33]. Due to the high prevalence of colorectal cancers in Saudi Arabian population, we examined four commonly studied polymorphic loci, rs1544410 (BsmI), rs2228570 (FokI), rs7975232 (ApaI) and rs731236 (TaqI) in the VDR gene for a possible association with the risk of developing this malignancy. The departure from Hardy-Weinberg equilibrium in this study for the SNPs rs7975232 and rs731236 in the CRC patients and rs1544410 and rs7975232 in the control population may be due to the high rate of consanguineous marriages in Saudi society [34–35]. The observed deviation from the Hardy-Weinberg equilibrium for the mentioned SNPs are unlikely due to misclassification of the genotypes as they were identified by highly reliable DNA sequencing in both the directions. In this study, none of the SNPs were found to be significantly associated with CRC in the overall analysis for both the genotypic and allelic models. The lack of association of these SNPs does not rule out the possibility of the involvement of VDR as other polymorphic loci in the gene might influence the development of CRC. In the absence of association of the VDR polymorphisms observed in our study, the increased incidence of CRCs may be attributed to the high prevalence of vitamin D deficiency among Saudis, since substantial evidence suggests that vitamin D may play significant role in CRC risk [36–38]. A recent study in a Jordanian population which may be ethnically similar to Saudi Arabians found a significant association between low serum level of vitamin D and CRCs. The authors also observed an association of VDR-TaqI polymorphism and CRC risk in Jordanians [39]. The discrepancy for the VDR-TaqI polymorphism between the Jordanian and Saudi population is not known and it is possible that certain confounding factors might be involved in the different results obtained for this genetic variant. Analysis after stratification of the samples based on age and gender suggests statistically significant association of CRC with the ‘C’ allele of rs7975232 in patients **>**57 years of age at disease diagnosis and rs1544410 in females. These results, however, needs further validation in larger cohorts due to the limitation of small sample size in our study. The 4.6 fold protective effect of the ‘AA’ genotype of rs1544410 compared to the ‘GG’ genotype in females could be due to the higher serum levels of 1,25(OH)_2_D_3_ associated with the ‘AA’ genotype relative to the ‘GG’ genotype [40]. Our results are in accordance with the findings of Takeshige and colleagues who also did not find significant association of colorectal cancers with any of the four VDR SNPs (BsmI, FokI, ApaI and TaqI) in Japanese patients [33]. Similarly, in a multivariate analysis adjusted for ethnicity, sex, age, body mass index, and noncancer status with polyps in African American and Hispanic populations, none of the four VDR SNPs were significantly associated with CRC. However, in a univariate analysis, while BsmI, ApaI and TaqI SNPs were not found to be associated with CRCs, the authors were able to observe significant association between VDR-FokI polymorphism and CRC cases [31]. Laczmanska et al, in a cohort of Polish colorectal cancer patients, did not find any association for the FokI polymorphism, however, the authors observed statistically significant correlation with the risk of CRC for the BsmI, ApaI and TaqI polymorphic loci [32]. A large meta-analysis on the VDR polymorphisms and cancer risk suggests only BsmI SNP to be associated with CRC in Caucasian population while the other three SNPs, FokI, ApaI and TaqI were not associated with CRCs [41]. Two other meta-analysis on the VDR-BsmI polymorphism also found significant association of this SNP with CRC [42–43]. Our analysis revealed VDR-BsmI polymorphism to be significantly associated with CRC in female patients, thus validating to some extent the findings from earlier studies. Gandini and colleagues reviewed 79 studies involving more than 52,000 different cancer cases and 62,000 controls to identify relevance of VDR BsmI, FokI, ApaI and TaqI polymorphism for various cancers and found BsmI, FokI and TaqI to be associated with CRC [27]. While we observed the ‘C’ allele of VDR-ApaI to confer 77% increased risk for CRC in patients **>**57 years of age at the time of disease diagnosis, a recent meta-analysis by Serrano et al did not identify higher risk of CRC with VDR-ApaI but found a significant association with VDR-TaqI polymorphism [44]. Several other studies did not find an association of these VDR genetic variants with colorectal cancer risk in Caucasian populations [28–30]. The discrepancies with the risk association of the VDR polymorphisms and colorectal tumors in various investigations could be due to the different genetic background of cancers where haplotype clusters might have more relevance rather than individual genotype. Ethnicity and environmental factors may also have considerable modifying effect in combination with the genetic variants towards the risk of the disease. We found significant differences in the distribution of the genotype frequencies of the VDR SNPs between Saudi Arabian population and several of the International HapMap project populations. This can in part explain the lack of association of the VDR SNPs in the overall analysis in this study compared to those who observed significant association with colorectal cancers in their populations.

The strength of the present study is that the cases and controls were from the same ethnicity and were age and gender matched. We also used a highly reliable DNA Sanger sequencing technique for detecting the genotypes of the study subjects. Limitations of this study included small sample size which may not be representative distribution of the population and hence results should be interpreted with caution without generalization. Moreover, the power to detect small effects is also likely to be decreased with smaller sample size. Although control subjects were age and gender matched, hospital-based enrollment of the participants may introduce selection bias and might be different from the population-based controls. Further, our study did not take in to account potential effect of environmental factors such as smoking, diet and exercise on the association of VDR polymorphisms and CRC risk.

In conclusion, this is the first study to assess the role of VDR polymorphism in the risk of developing colorectal cancer in Saudi Arabian population. While in the overall analysis the VDR-BsmI, FokI, ApaI and TaqI SNPs did not correlate with the susceptibility to CRC, ApaI and BsmI was significantly associated with CRC in elderly and female patients, respectively. These findings need to be validated in larger cohorts of Saudi ethnicity to predict any beneficiary role of utilizing these VDR polymorphisms as a potential screening marker for colorectal cancers in our population.
